# 0030. Effect of exercise training on muscle function in a recovery model of critical illness

**DOI:** 10.1186/2197-425X-2-S1-O4

**Published:** 2014-09-26

**Authors:** A Sigurta, S Saeed, M Singer

**Affiliations:** University College London, Bloomsbury Institute of Intensive Care Medicine, London, UK

## Introduction

Survivors of critical illness experience significant skeletal muscle weakness and physical disability, which may persist for years [1]. Early mobilization is being encouraged to improve functional outcomes [2]. Decreased mitochondrial function and altered mitochondrial biogenesis are implicated in the pathogenesis of sepsis-induced muscle dysfunction [3]. Mitochondrial biogenesis can be stimulated by physical activity [4].

## Objectives

To assess the role of exercise training on muscle function and mitochondrial biogenesis in a long term rat model of critical illness and recovery.

## Methods

Peritonitis was induced in male Wistar rats by i.p. injection of the fungal cell wall product, zymosan. Animals were divided into 2 groups: (i) trained animals who underwent daily motorised treadmill sessions from day 2-14, progressively increasing treadmill speed and duration to 30 mins at 30 cm/s; (ii) control animals.

Weight and clinical score were recorded daily. Muscle function was assessed on days 2, 7 and 14 using exercise capacity and forelimb grip strength. On day 14, animals were culled for harvesting of gastrocnemius and soleus muscle that were weighed and then used to measure (by RT-PCR) gene expression assays of the biogenesis factors, PGC-1alpha, NRF and Tfam. Results given as ratios, using HMBS as the housekeeping gene.

## Results

**Figure 1:**
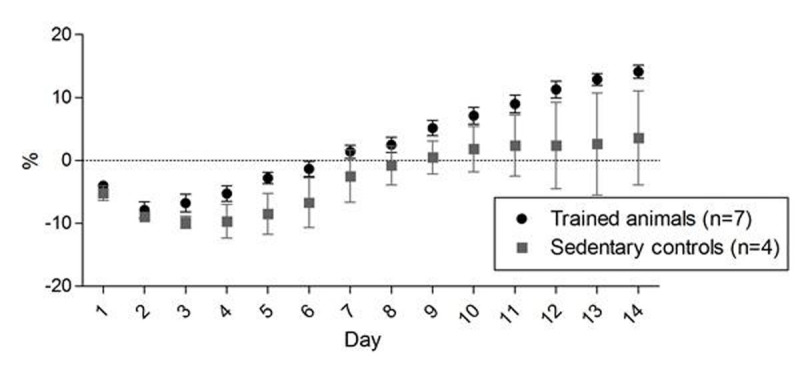
Changes in body weight expressed as percentage from baseline weight. Data shown as mean ± SE.

**Figure 2:**
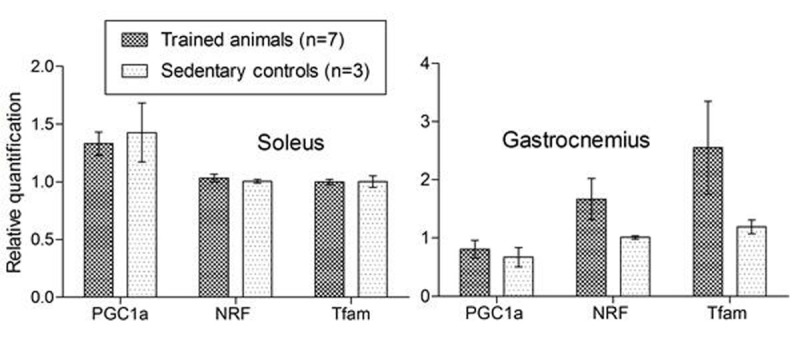
Real-time PCR (mean ± SE) on Day 14 soleus and gastrocnemius muscle.

All animals lost weight post-zymosan and gained weight thereafter, with trained animals doing so at a greater rate (Fig 1). Exercise capacity increased by approximately 50% in both groups between Day 2 and Day 7, but was only maintained at Day 14 in trained animals. Grip strength was maintained throughout in trained animals but fell by 29.2±14% at Day 7 in controls. Tfam and NRF, but not PGC-1alpha, were higher in gastrocnemius in trained animals, but not in soleus (Fig 2).

## Conclusions

Exercise training increases weight gain in this model of critical illness and recovery. Preliminary data shows improved mitochondrial biogenesis in gastrocnemius but not soleus with exercise.
